# A food plant specialist in Sparganothini: A new genus and species from Costa Rica (Lepidoptera, Tortricidae)

**DOI:** 10.3897/zookeys.303.5230

**Published:** 2013-05-21

**Authors:** John W. Brown, Daniel H. Janzen, Winnie Hallwachs

**Affiliations:** 1 Systematic Entomology Laboratory, Agricultural Research Service, U.S. Department of Agriculture, National Museum of Natural History, P.O. Box 37012, Washington, DC 20013–7012, USA; 2 Department of Biology, University of Pennsylvania, Philadelphia, 19104, USA; 3 Department of Biology, University of Pennsylvania, Philadelphia, 19104, USA

**Keywords:** ACG, caterpillar, Costa Rica, Cyclanthaceae, food plants, morphology, new genus, new species, parasitoid, tortricid moth

## Abstract

*Sparganocosma docsturnerorum* Brown, new genus and new species, is described and illustrated from Área de Conservación (ACG) in northwestern Costa Rica. The new genus shares a long, crescent- or ribbon-shaped signum in the corpus bursae of the female genitalia with *Aesiocopa* Zeller, 1877, *Amorbia* Clemens, 1860, *Amorbimorpha* Kruse, 2011, *Coelostathma* Clemens, 1860, *Lambertiodes* Diakonoff, 1959, *Paramorbia* Powell & Lambert, 1986, *Rhynchophyllus* Meyrick, 1932, *Sparganopseustis* Powell & Lambert, 1986, *Sparganothina* Powell, 1986, and *Sparganothoides* Lambert & Powell, 1986. Putative autapomorphies for *Sparganocosma* include the extremely short uncus; the smooth (unspined) transtilla; and the upturned, free, distal rod of the sacculus. Adults of *Sparganocosma docsturnerorum* have been reared numerous times (>50) from larvae collected feeding on rain forest *Asplundia utilis* (Oerst.) Harling and *Asplundia microphylla* (Oerst.) Harling (Cyclanthaceae) at intermediate elevations (375–500 m) in ACG. Whereas most Sparganothini are generalists, typically feeding on two or more plant families, *Sparganocosma docsturnerorum* appears to be a specialist on *Asplundia*, at least in ACG. The solitary parasitoid wasp *Sphelodon wardae* Godoy & Gauld (Ichneumonidae; Banchinae) has been reared only from the larvae of *Sparganocosma docsturnerorum*.

## Introduction

With over 10,000 described species worldwide, Tortricidae are among the largest families of microlepidoptera (Regier et al. 2012). Their economic importance as pests of forests, ornamentals, and crops; their successful application as biological control agents against undesired invasive plants; and their use as “model systems” (e.g., Roe et al. 2009) all combine to attract considerable attention (Regier et al. 2012). Over the last decade, our taxonomic knowledge of the family likely has increased faster than that of any other microlepidoptera family, with an average of about 13 new genera and 200 new species described per year (Brown 2012).

Within Tortricidae the tribe Sparganothini has been the subject of substantial recent monographic work, with systematic treatments of *Sparganothina* Powell, 1986, and relatives by Landry and Powell (2001); *Amorbia* Clemens, 1860 by Phillips-Rodriguez and Powell (2007); *Spaganothoides* Powell and Lambert, 1986, by Kruse and Powell (2009); and *Amorbimorpha* Kruse, 2011, by Kruse (2011); and a review of the entire North American fauna by Powell and Brown (2012). Whereas a stable generic-level classification is in place for the North American members, many described and undescribed species from the Neotropics defy confident generic assignment. The purpose of this contribution is to describe a new genus and species from Costa Rica to continue to build on the growing generic framework for the tribe.

## Methods

Rearing. During an ongoing survey of the Lepidoptera of Área de Conservación Guanacaste in northwestern Costa Rica (Janzen et al. 2009), adults and caterpillars have been collected throughout the year. Larvae discovered in the field are taken to “rearing barns” where they are placed individually in plastic bags with cuttings of the food plant on which they were discovered. As adults emerge, they are dispatched by freezing, then pinned and labeled. Each specimen receives a unique voucher number in the form of YY-SRNP-XXXX (e.g., 09-SRNP-15328), where the prefix is the last two digits of the year (e.g., 2009), “SRNP” refers to the project “call letters” assigned in 1977 (when the project site was referred to as Santa Rosa National Park), and the suffix is a unique number assigned within the year.

Morphology. Dissection methods follow those presented in Brown and Powell (1991). Images ofadults and genitalia werecaptured usingaCanon EOS 40D digital SLR (Canon U.S.A., Lake Success, NY) mounted on a Visionary Digital BK Lab System (Visionary Digital, Palmyra, VA). Terminology for genitalia structures and forewing pattern elements follows Powell and Brown (2012). In descriptions of the forewing, “dorsum” refers to the hind margin of the forewing, which is the dorsal-most edge of the wings when the live moth is in resting posture and the forewings are held in a tent-like position over the abdomen.

Depositories and Abbreviations. The holotype of the new species is deposited in the National Museum of Natural History, Washington, D.C., U.S.A. Paratypes are deposited in the Canadian National Collection of Insects, Ottawa, Canada; Instituto Nacional de Biodiversidad, Santa Domingo de Heredia, Costa Rica; The Natural History Museum, London, England; Essig Museum of Entomology, University of California, Berkeley, U.S.A.; and National Museum of Natural History, Washington, D.C., U.S.A. Abbreviations used in text are as follows: ACG = Área de Conservación Guanacaste; ec = eclosed; r.f. = reared from.

## Results

### Sparganocosma

Brown gen. n.

urn:lsid:zoobank.org:act:67B1DA19-F8BF-4D49-A6AC-E86CCFACB618

http://species-id.net/wiki/Sparganocosma

#### Type species.

*Sparganocosma docsturnerorum* Brown, new species.

#### Diagnosis.

In facies, *Sparganocosma* are unlike any other known sparganothine genus. The forewing pattern is somewhat two-toned longitudinally, pale buff along the costal half, usually interrupted near the middle of the wing by an ill-defined area of darker scales, and dark brown along the dorsal half, broadening toward the termen and apex. The forewing also has a characteristic long, slender costal fold in the male. Adults are about the same size (forewing length) or slightly larger than *Amorbia* Clemens, 1860 and *Aesiocopa* Zeller, 1877 with similar sexual dimorphism in size – females are slightly larger than males. Dimorphism in forewing pattern in *Sparganocosma* is less pronounced than in *Aesiocopa* and slightly more pronounced than in *Amorbia*. The labial palpi in *Sparganocosma* are similar to those of many *Amorbia* – somewhat upturned-porrect, their combined (all three segments) length 2.2–2.5 times the diameter of the compound eye, and with little sexual dimorphism. In contrast, the labial palpi of most Sparganothini are conspicuously long and porrect and frequently exhibit pronounced sexual dimorphism (see Powell and Brown 2012). Abdominal dorsal pits are absent in *Sparganocosma*, whereas they are present in *Aesiocopa*, many species of *Amorbia*, and a few other sparganothine genera (e.g., *Coelostathma*, *Sparganopseustis*).

In the female genitalia, the signum of *Sparganocosma* is broad and band shaped, similar to that of several other sparganothines (i.e., *Aesiocopa*, *Amorbia*, *Amorbimorpha*, *Coelostathma* Clemens, 1860, *Lambertiodes* Diakonoff, 1959, *Paramorbia* Powell & Lambert, 1986, *Rhynchophyllus* Meyrick, 1932, *Sparganopseustis* Powell & Lambert, 1986, *Sparganothina*, and *Sparganothoides*), but it is distinguished from that of other genera by its slightly rounded-triangular swelling in the middle, which is unique to *Sparganocosma*. The sterigma in *Sparganocosma* is weakly bilobed, and therefore similar to that of many *Amorbia* and *Coelostathma*.

In the male genitalia of *Sparganocosma* the secondary arms of the socius are absent, a character state shared with *Amorbia*, *Paramorbia*, *Sparganothina*, and *Coelosthathma*. The male genitalia are distinguished from those of all other Sparganothiini by the extremely short uncus (approximately 0.3 the length of the socius); the smooth (lacking spines), slender transtilla; and the long, upturned, free distal rod of the sacculus. The latter is reminiscent of that found in some species of *Sparganothina*, but the two genera are extremely dissimilar in forewing size and maculation.

#### Description.

**Figures 1–6.**
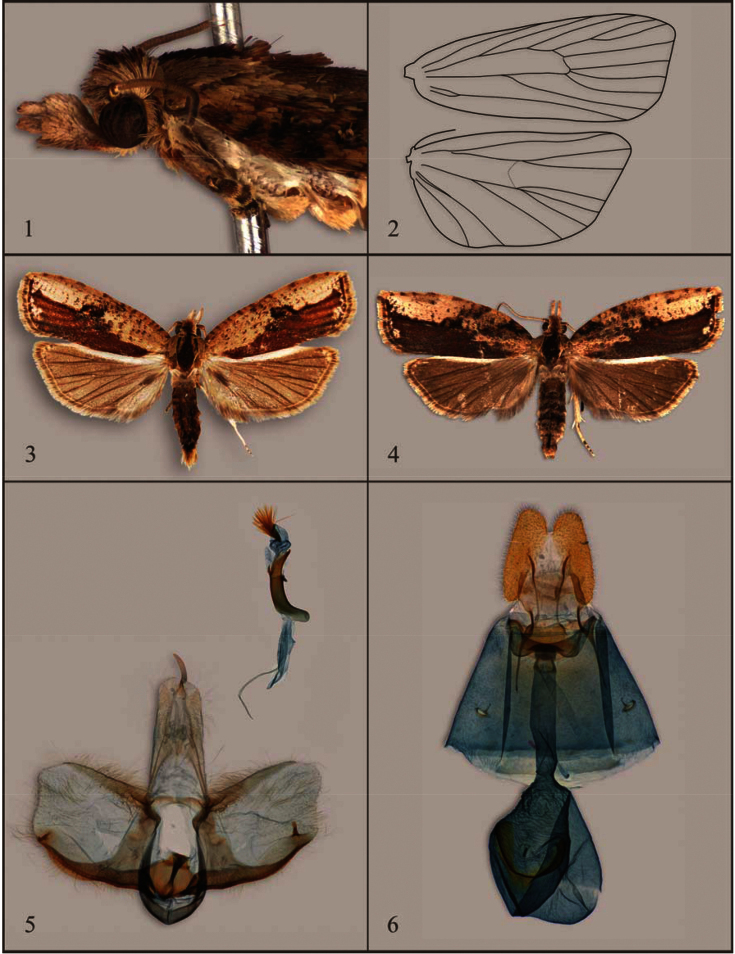
Features of the adult of *Sparganocosma docsturnerorum*. **1** Head of female (06-SRNP-42632) **2** Wing venation of male (01-SRNP-23411.11) **3** Holotype male (04-SRNP-40557) **4** Paratype female (04-SRNP-40558) **5** Male genitalia; USNM slide 142,039 (04-SRNP-42252) **6** Female genitalia; USNM slide 142,040 (04-SRNP-42248).

Head: Vertex rough scaled with overhanging tuft, upper frons rough scaled, lower frons smooth scaled, without complex hood. Labial palpus (Figure 1) moderate in length, segment II 1.5–1.8 times horizontal diameter of compound eye, weakly upcurved; segment III exposed, porrect. Ocellus minute or inconspicuous. Antennal scaling in two bands per segment, sensory setae 0.7–0.8 times flagellomere width in male, shorter, sparser in female. Thorax: Tegula large, nota smooth scaled; legs unmodified. Forewing (Figure 2) broad, about 2.6 times as long as wide, with narrow costal fold in male, extending ca. 0.4 length of costa; no raised scales present; all veins present and separate, except R_4_ and R_5_ stalked in basal 0.35–0.40 in both sexes, with both extending to costa before apex; chorda and m-stem absent. Hindwing with Rs and M_1_ approximate at base, CuA_1_ and M_3_ connate, and M_2_ and M_3_ approximate at base; cubital hair pecten well developed in both sexes. Abdomen: Dorsal pits absent. Female lacking enlarged corethrogyne scaling. Male genitalia with uncus small, approximately 0.3 times length of socius, weakly curved ventrad; socius slender, slightly broadened posteriorly, densely clothed in long scales, mostly fused to tegumen, but with free, membranous posterior lobe, lacking secondary arm; gnathos absent; transtilla short, smooth (lacking spines), slightly arched medially; pulvinus weakly developed, represented by basal termination of linear patch of setae along costa of valva; valva broad, short, mostly parallel-sided, with concave “notch” apically (similar to *Amorbia*); row of strong setae along subcosta, except basally; sacculus well defined, weakly undulate, with a long, free rod near termination. Phallus approximately 0.75 length of valva, curved at about 135° angle at approximately 0.3 distance from base, with small dorsal spur in distal 0.3; vesica with dense bundle of 40–50 aciculate, subbasally attached, deciduous cornuti. Female genitalia with papillae anales, simple, unmodified, slightly narrowed anteriorly; apophyses simple, about as long as papillae anales, posteriores only slightly longer than anteriores; sterigma a shallow, weakly bilobed bowl, with conspicuous subcircular sclerite in postostial sterigma; ostium defined posteriorly by narrow, strongly scleritozed ridge; ductus bursae relatively broad throughout, slightly longer than corpus bursae, with short, well defined but membranous colliculum; corpus bursae round or slightly bilobed, densely and finely wrinkled; signum a broad, curved ribbon with small, rounded-triangular expansion near middle, truncate at each end, situated in anterior half of corpus bursae; tiny, semi-membranous, knob-like process on exterior surface of corpus bursae near signum (as in *Amorbia* and *Aesiocopa*).

#### Etymology.

The genus name is from the Latin “spargano,” to scatter or throw around, and “cosm,” referring to the universe. It is interpreted as masculine.

### Sparganocosma docsturnerorum

Brown sp. n.

urn:lsid:zoobank.org:act:C01718B4-16CF-431E-AA0B-798E25C11401

http://species-id.net/wiki/Sparganocosma_docsturnerorum

Figures 1 –8

#### Diagnosis.

*Sparganocosma docsturnerorum* can be distinguished from all other Sparganothini, and from all other Tortricidae, by the characters discussed in the diagnosis of the genus above. The distinctive forewing pattern easily distinguishes it from all other Sparganothini, and it is further differentiated by unique features of the male genitalia and female.

#### Description.

Head: Vertex pale buff with variably developed patch of pale maroon medially; frons and labial palpus slightly lighter pale buff. Antenna mostly pale buff, except scape maroon. Thorax: Tegula pale buff, nota maroon, except pale cream along narrow lateral margins. Legs mostly pale brown with narrow pale-yellow banding. Forewing (Figures 3, 4) length 8.8–11.0 mm (mean = 9.9; n = 10) in male, 10.5–13.0 mm (mean 11.6; n = 10) in female; forewing with two large ovoid patches of pale buff in costal region, one from base to approximately 0.5 distance to apex, the other in distal 0.45, basal patch less defined in female, irregularly overscaled with brown; patches infrequently separated by narrow brown remnant of median fascia; patches usually with small flecks of brown; remainder of wing with broad brown longitudinal band along dorsum, narrowest at base, broadest at termen, with faint traces of pale buff along veins in distal part of wing or with tiny spots of pale buff near wing margin; longitudinal band along dorsum infrequently paler or lacking altogether in male. Fringe pale buff. Hindwing rather uniformly dark gray brown, slightly darker in female. Fringe pale cream gray. Abdomen: Pale brownish gray. Male genitalia (Figure 5) as described above for genus. Female genitalia (Figure 6) as described above for genus.

Holotype, ♂, Costa Rica, Alajuela Province, Área de Conservación Guanacaste, Sector Rincón Rain Forest, Río Francia Arriba, 400 m, 10.89666N, 85.29003W, 24 Feb 2002, r.f. *Asplundia utilis*, José Pérez; ec: 29 Mar 2004 (04-SRNP-40557).

Paratypes (32♂, 41♀). COSTA RICA: **Alajuela Province**: Área de Conservación Guanacaste: Sector Rincón Rain Forest: Sendero Anonas, 405 m, 10.90528N, 85.27882W, 23 Nov 2001, r.f. *Asplundia utilis*, José Pérez, ec: 28 Dec 2001 (1♀) (01-SRNP-23411); ec: 29 Dec 2001 (1♀) (01-SRNP-23411.01); ec: 30 Dec 2001 (1♂) (01-SRNP-23411.02); ec: 29 Dec 2001 (1♂) (01-SRNP-23411.04); ec: 30 Dec 2001 (1♀) (01-SRNP-23411.07); ec: 30 Dec 2001 (1♀) (01-SRNP-23411.09); ec: 30 Dec 2001 (1♂) (01-SRNP-23411.11); ec: 27 Dec 2001 (1♀) (01-SRNP-23411.12); ec: 29 Dec 2001 (1♀) (01-SRNP-23411.19); ec: 20 Dec 2001 (1♀) (01-SRNP-23411.21); ec: 28 Dec 2001 (1♀) (01-SRNP-23411.22); ec: 29 Dec 2001 (1♂) (01-SRNP-23411.26); ec: 29 Dec 2001 (1♀) (01-SRNP-23411.27); ec: 30 Dec 2001 (1♀) (01-SRNP-23411.28); ec: 28 Dec 2001 (1♀) (01-SRNP-23411.31); 7 Nov 2011, A. Córdoba, ec: 9 Dec 2011 (1♂), r.f. *Asplundis utilis* (11-SRNP-44797). Jacobo, 461 m, 10.94076N, 85.3177W, 19 May 2011, Calixto Moraga, ec: 10 Jun 2011 (3♀), ec: 9 Jun 2011 (3♂), r.f. *Asplundis utilis* (11-SRNP-80462, 11-SRNP-80464, 11-SRNP-80449, 11-SRNP-80457, 11-SRNP-80459, 11-SRNP-80448). Sendero Rincón, 430 m, 10.8962N, 85.27769W, 18 Aug 2000, r.f. *Asplundia utilis*, ec: 9 Sep 2000 (1♂) (00-SRNP-14214); ec: 12 Sep 2000 (1♀) (00-SRNP-14216). Sendero Rincón, 430 m, 10.8962N, 85.27769W, 15 Feb 2006, r.f. *Asplundia utilis*, Minor Carmona, ec: 13 Mar 2006 (1♀) (06-SRNP-40592), ec: 14 Mar 2006 (1♀) (06-SRNP-40590), ec: 14 Mar 2006 (1♀) (06-SRNP-40591). Vado Río Francia, 400 m, 10.90093N, 85.28915W, 20 Feb 2002, r.f. *Asplundia utilis*, José Pérez, ec: 18 Apr 2002 (1♂) (02-SRNP-6476); ec: 24 Mar 2004 (1♂) (02-SRNP-6474). Río Francia Arriba, 400 m, 10.89666N, 85.29003W, 24 Feb 2002, r.f. *Asplundia utilis*, José Pérez; ec: 29 Mar 2004 (1♂) (04-SRNP-40555); ec: 30 Mar 2002 (1♂) (04-SRNP-40556); ec: 30 Mar 2004 (1♀) (04-SRNP-40553); 30 Mar 2004 (1♀) (04-SRNP-40558); 24 May 2004, r.f. *Asplundia utilis*, José Pérez, ec: 28 Jun 2004 (1♀) (04-SRNP-41311); 25 Oct 2011, A. Córdoba, ec: 4 Dec 2011 (1♀), r.f. *Asplundis utilis* (11-SRNP-44698); 18 Mar 2011, A. Córdoba, ec: 12 Apr 2011 (1♂), 13 Apr 2011 (1♀), r.f. *Asplundis utilis* (11-SRNP-41257, 11-SRNP-41260); 7 Oct 2010, Pablo Calderón, ec: 17 Nov 2010 (1♂), ec: 19 Nov 2010 (1♀), r.f. *Asplundis utilis* (10-SRNP-43639, 10-SRNP-43637); 18 Mar 2011, A. Córdoba, ec: 12 Apr 2011 (1♂, 1♀), r.f. *Asplundis utilis* (11-SRNP-41261, 11-SRNP-41259). Montanya Figueres, 460 m, 10.88367N, 85.29081W, 22 Oct 2009, r.f. *Asplundia utilis*, Pablo Umaña, ec: 29 Nov 2009 (1♂) (09-SRNP-43035). Finca Aurita, 460 m, 10.88409N, 85.25728W, 4 Jan 2007, r.f. *Asplundia utilis*, José Pérez, ec: 1 Feb 2007 (1♂) (07-SRNP-40058); ec: 3 Feb 2007 (1♀) (07-SRNP-40050), ec: 2 Feb 2007 (1♀) (07-SRNP-40045). Finca Aurita, 460 m, 10.88409N, 85.25728W, 23 Nov 2006, r.f. *Asplundia utilis*, José Pérez, ec: 2 Jan 2007 (1♀) (06-SRNP-44494). Quebrada Guarumo, 400 m, 10.90445N, 85.28412W, 24 Jul 2006, r.f. *Asplundia utilis*, José Pérez, ec: 1 Sep 2006 (1♂) (06-SRNP-42634); ec: 1 Sep 2006 (1♀) (06-SRNP-42629); ec: 2 Sep 2006 (1♀) (06-SRNP-42628); ec: 31 Aug 2006 (1♀) (06-SRNP-42631); ec: 1 Sep. 2006 (1♀) (06-SRNP-42632); 3 Mar 2011, A. Córdoba, ec: 11 Apr 2011 (1♂),ec: 10 Apr 2011 (1♀), r.f. *Asplundis utilis* (11-SRNP-41108, 11-SRNP-41109). Sendero Parcelas, 375 m, 10.90777N, -85.29137, 26 Aug 2004, r.f. *Asplundia utilis*,José Pérez, ec: 23 Sep 2004 (1♂) (04-SRNP-42252), 25 Sep. 2004 (1♂) (04-SRNP-42253), 25 Sep 2004 (1♂) (04-SRNP-42251), 26 Sep. 2004 (1♂) (04-SRNP-42254), ec: 28 Sep 2004 (1♀) (04-SRNP-42248), ec: 7 Sep 2004 (1♀) (04-SRNP-42249), 29 Sep 2004 (1♀) (04-SRNP-42250). Quebrada Escondida, 420 m, 10.89928N, 85.27486W, 4 Mar 2002, r.f. *Asplundia utilis*, ec: 27 Mar 2002 (1♀) (02-SRNP-6614), ec: 27 Mar 2002 (1♀) (02-SRNP-6613). Camino Porvenir, 383 m, 10.90383N, 85.25964W, 5 Feb 2007, r.f. *Asplundia utilis*, Minor Carmona, ec: 3 Mar 2007 (1♀) (07-SRNP-40382). Sendero Juntas, 400 m, 10.90661N, 85.28784W, 21 Jan 2007, r.f. *Asplundia utilis*, Minor Carmona, ec: 1 Mar 2007 (1♀) (07-SRNP-40231). **Guanacaste Province**: Sector San Cristobal: Río Blanco Abajo, 500 m, 10.90037N, 85.37254W, 12 Dec, 2011, C. Cano, ec: 8 Jan 2012 (2♂), ec: 9 Jan 2012 (1♂), ec: 12 Jan 2012 (1♂), ec: 9 Jan 2012 (1♂), r.f. *Asplundia microphylla* (11-SRNP-4889, 11-SRNP-4899, 11-SRNP-4891, 11-SRNP-4904, 11-SRNP-4903). Rio Blanco Abajo, 500 m, 10.90037N, 85.37254W, 12 Dec 2011, r.f. *Asplundia microphylla*, Carolina Cano, ec: 10 Jan 2012 (2♂) (11-SRNP-4882, 11-SRNP-4888); ec: 8 Jan 2012 (1♂) (11-SRNP-4886). Sector Pitilla, Quebradona, 475 m, 10.99102N, 85.39539W, 21 May 2011, Ricardo Calero, ec: 5 Jun 2011 (1♀), r.f., unknown plant (11-SRNP-71121).

**Figures 7–8.**
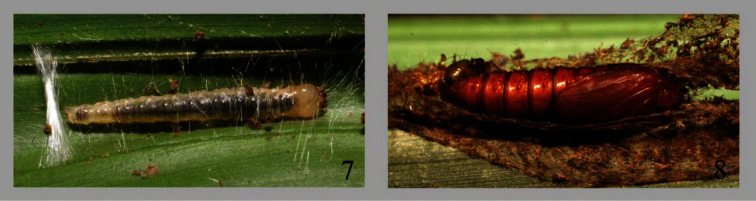
Early stages of *Sparganocosma docsturnerorum*. **7** Penultimate instar larva **8** Pupa.

#### Distribution and biology.

*Sparganocosma docsturnerorum* is known only from this one small area of Costa Rica, despite intensive moth collecting throughout Costa Rica by Janzen, Hallwachs, the INBio parataxonomists, and visiting scientists for over 30 years. The entire type series (n = 53) was reared from larvae collected while they were feeding on *Asplundia utilis* (Oerst.) Harling and *Asplundia microphylla* (Oerst.) Harling (Cyclanthaceae) growing in the heavily shaded rain forest understory at intermediate elevations (375–500 m) in ACG. Although some rearing records previously reported the food plant as *Carlodovica costaricensis* (Cyclanthaceae), this name is currently considered a synonym of *Asplundia utilis* (Williams 1961), and the project databases have been updated accordingly. With exceedingly few documented exceptions, species of Sparganothini are moderately to highly polyphagous (Powell and Brown 2012), typically feeding on two or more plant families. Hence, it is interesting that *Sparganocosma docsturnerorum* has been reared from a single plant genus in ACG, suggesting a high degree of host plant specialization. Of course, this does not preclude the possibility that it feeds on other plants in other parts of its range, wherever that may be. The range of this moth will be difficult to determine given its apparent lack of attraction to lights.

Superficially, the penultimate instar of *Sparganocosma docsturnerorum* is pale translucent yellow-gold, with fine, long, pale setae from unmarked pinacula (Figure 7). The head is nearly uniformly amber with a small black spot in the stemmatal area. The pupa is typically tortricoid (Figure 8), with two rows of spines on the dorsum of abdominal segments 3–9, and lacks dorsal pits. Development time from prepupa to eclosion required 21–25 days. In the course of the ACG caterpillar inventory through 2011, 223 larvae of *Sparganocosma docsturnerorum* have been collected and reared. From these rearings have emerged 13 solitary parasitoid wasps, *Sphelodon wardae* Godoy & Gauld (Ichneumonidae; Banchinae), the host of which was formerly unknown (Godoy & Gauld 2002). This wasp oviposits in the larva and exits from the prepupal larva inside the moth’s cocoon, where it spins its own flimsy cocoon. In the entire ACG caterpillar inventory, about 510,000 wild-caught caterpillars have yielded about 52,000 parasitoid records, of which the 13 records of *Sphelodon wardae* have come only from *Sparganocosma docsturnerorum*, along with four records of an undescribed parasitoid fly (*Actia*, Tachinidae) from the same sample of moth larvae. If *Sphelodon wardae* is a specialist on *Sparganocosma docsturnerorum* as the data suggest, then the geographic distribution of the moth likely includes the localities from which the parasitoid has been recorded – the provinces of Limón, Cartago, Guanacaste, and Heredia, at elevations between 400 and 1000 m.

The near absence of field-collected adults (we examined one genitalia slide of a presumably light-collected specimen from the OET Estación Biologica La Selva, Heredia, Costa Rica, but could not locate the associated adult) suggests that this species is not attracted to light, especially since light-trapping has been conducted on many nights during the Lepidoptera inventory of this ACG rain forest ecosystem (Janzen et al. 2009). A similar phenomenon is observed in *Aesiocopa* where the vast majority of specimens has been either reared or collected from malaise traps (Brown in press). In contrast, virtually all species of *Amorbia*, *Sparganothoides*, *Coelostathma*, *Platynota*, and other ACG sparganothines are frequently encountered at lights and/or collected in light traps.

#### Etymology.

The specific epithet is a patronym for Drs. John Turner and Nancy Turner of Ardmore, Tennessee, USA, whose intense curiosity about tropical Lepidoptera in general, and Riodinidae specifically, has psychologically and financially strongly supported the Lepidoptera inventory of ACG.

## Discussion

Relationships among sparganothine genera have not been investigated in a modern phylogenetic context, so the position of *Sparganocosma* within the tribe cannot be determined with certainty. The long, crescent- or ribbon-shaped signum in the corpus bursae of the female genitalia of *Sparganocosma* may represent a synapomorphy for a sparganothine clade that includes *Aesiocopa*, *Amorbia*, *Amorbimorpha*, *Coelostathma*, *Lambertiodes*, *Paramorbia*, *Rhynchophyllus*, *Sparganopseustis*, *Sparganothina*, and *Sparganothoides*. The presence of secondary arms of the socii divide the group – they are present in *Aesiocopa*, *Amorbimorpha*, *Sparganopseustis*, and *Sparganothoides* and absent in *Amorbia*, *Coelostathma*, *Lambertiodes*, *Paramorbia*, *Sparganothina*, and *Sparganocosma*. The male of *Rhynchophyllus* is unknown. Within the later group of genera, *Sparganocosma* lacks abdominal dorsal pits, which are present in many *Amorbia* and nearly all *Coelostathma* (Phillips-Rodriguez & Powell 2007; Powell & Brown 2012).

Based on DNA barcode data (cytochrome oxidase 1) (i.e., Janzen et al. 2009), specimens of *Sparganocosma docsturnerorum* (n = 31) form a tight cluster with exceedingly limited genetic divergence (less than 0.5%) among them. However, there is a suspicious shallow split in the cluster of barcodes portrayed in a CO1 neighbor joining (NJ) tree, a split that is correlated with collection site. Hence, the data and specimens require further scrutiny since other species of ACG Lepidoptera with no more barcode distance among them have turned out to be species complexes (e.g., Burns et al. 2007, 2008). In NJ trees, the genus is portrayed as near *Sparganothoides*, *Coelostathma*, and *Paramorbia*. However, such trees should be used primarily as an aid to determining species boundaries and for the discovery of cryptic species, not as an indication of phylogenetic relationships because mitochondrial DNA data are subject to biases that may obscure true phylogenetic signal (e.g., Will & Rubinoff 2004; Rubinoff et al. 2006).

## Supplementary Material

XML Treatment for Sparganocosma

XML Treatment for Sparganocosma docsturnerorum
